# Supplementary material for: RNR-R2 Upregulation by a Short Non-Coding Viral Transcript
Source: Biomolecules. 2021 Dec 3;11(12):1822. doi: 10.3390/biom11121822 (PMC8698843; doi:10.3390/biom11121822)
Supplement: Supplementary file 1 [file biomolecules-11-01822-s001.zip › biomolecules-1486006-supplementary.pdf]

biomolecules

MDPI

Supporting Information

Hepatitis B virus RNA plays a non-coding role in upregulating RNR-R2 expression

Karin Broennimann, Inna Ricardo-Lax, Julia Adler, Eleftherios Michailidis,

Ype P de Jong, Nina Reuven and Yosef Shaul

Table of Contents

Plasmid list:..2

Primer sequences……………………………………………………………………........5

Antibodies……………………………………………………………………………....6

Supplementary figures

Figure S1:...7

Figure S2..8

Figure S3..10

Figure S4..11

Figure S5..12

Figure S6:..………...13

Figure S7..14

Figure S8..16

Plasmid List

Plasmid Name	Plasmid Backbone	Location of Insert	Insert
Empty vector	pLenti4 (Invitrogen)	Gateway LR sites	No insert
HBx (full length)	pLenti4 (Invitrogen)	Gateway LR sites	HA-tag 5’<br>HBx (nucleotides 1374-1838)<br>HBV 3’UTR (nucleotides 1838-1989 genotype A, adw2)
HBx G27 stop	pLenti4 (Invitrogen)	Gateway LR sites	HA-tag 5’<br>The nucleotides 79-81 GGA (G) are mutated to the stop codon TGA
HBx ATG -> TTG	pLenti4 (Invitrogen)	Gateway LR sites	HA-tag 5’<br>A stop codon is inserted after the HA sequence. The ATG at position 1 is mutated to TTG.<br>HBV 3’UTR
HBx ATG -> TTG E80 stop	pLenti4 (Invitrogen)	Gateway LR sites	HA-tag 5’<br>A stop codon is inserted after the HA sequence. The ATG at position 1 is mutated to TTG. Nucleotides 235-237 are ATG. Nucleotides 238-240 of E80 were mutated from GAG to TAG (stop)<br>HBV 3’UTR
HBx ATG -> TTG S104 stop	pLenti4 (Invitrogen)	Gateway LR sites	HA-tag 5’<br>A stop codon is inserted after the HA sequence. The ATG at position 1 is mutated to TTG. Nucleotides 307-309 are ATG. Nucleotides 310-312 of S104 were mutated from TCA to TGA (stop).<br>HBV 3’UTR
no X ORF	pLenti4 (Invitrogen)	Gateway LR sites	HA-tag 5’<br>HBV 3’UTR
HBx 1374-1697	pLenti4 (Invitrogen)	Gateway LR sites	HA-tag 5’<br>HBx 1374-1697<br>HBV 3’UTR
HBx 1698-1838	pLenti4 (Invitrogen)	Gateway LR sites	HA-tag 5’<br>HBx 1698-1838<br>HBV 3’UTR
HBx 1463-1697	pLenti4 (Invitrogen)	Gateway LR sites	HA-tag 5’<br>HBx 1463-1697<br>HBV 3’UTR
HBx 1463-1571<br>Control RNA	pLenti4 (Invitrogen)	Gateway LR sites	HA-tag 5’<br>HBx 1463-1571<br>HBV 3’UTR
HBx 1572-1697<br>ERE	pLenti4 (Invitrogen)	Gateway LR sites	HA-tag 5’<br>1572-1697 (ERE)<br>HBV 3’UTR
HBx Δ3’UTR	pLenti4 (Invitrogen)	Gateway LR sites	HA-tag 5’<br>HBx
HBx ΔHA	pLenti4 (Invitrogen)	Gateway LR sites	HBx<br>HBV 3’UTR
HBx 1374-1838 mutated	pLenti4 (Invitrogen)	Gateway LR sites	HA-tag 5’<br>HBx sequence with the mutations shown in Fig S3<br>HBV 3’UTR
ERE no CMV	pLenti4 (Invitrogen)	Gateway LR sites	CMV promoter was removed<br>HA-tag 5’<br>1572-1697 (ERE)<br>HBV 3’UTR
Reverse ERE	pLenti4 (Invitrogen)	Gateway LR sites	Reverse complement of:<br>HA-tag 5’<br>1572-1697<br>HBV 3’UTR
HBx 1572-1697<br>ERE	pLenti4 (Invitrogen)	Gateway LR sites	HA-tag 5’<br>1572-1697<br>HBV 3’UTR
HBx 1572-1697<br>ERE	pLenti4 (Invitrogen)	Gateway LR sites	HA-tag 5’<br>1572-1697<br>HBV 3’UTR

HBV	pHR' CMV GFP<br>Inder Verma lab<br>Addgene plasmid # 14858 [17]	XmaI - XhoI	1.3x HBV<br>1.3x copies of the HBV adw2 strain from unique EcoRV site at its 5' end to the unique Taq1 site at its 3' end (position 1043 and 2017 relative to EcoRI restriction site) [18]
pSG5	pSG5 (Stratagene)		
pSG5 HBx	pSG5 (Stratagene)	NcoI - BglII	HA-tag 5'<br>HBx<br>3'UTR

qRT-PCR Primers

R2 fw 5'AGAGAGTAGGCGAGTATCAGAGG

rev 5'CAAGTAAGGGCACATCTTCAGTTC,

18S ribosomal RNA fw 5'TCGGAACTGAGGCCATGATTAAG

rev 5'CGGAACTACGACGGTATCTGATC

HBx 3'UTR fw 5'CATGTCCCACTGTTCAAGCC

rev 5'TCTGACGGAAGGAAAGAAGTCA

5'UTR (of pLenti4 (Gateway) based plasmids) fw 5'AAAAGCAGGCTTCGAAGGAGA

rev 5'CCACCCAAGCTAGCGTAATCT

HBx ORF fw 5'CCACCGTGAACGCCCATC

rev 5'TTGTGCCTACAGCCTCCTAATAC

HBs ORF fw 5'ACATCAGGATTCCTAGGACC

rev 5'TATCGCTGGATGTGTCTGCG

RPS11 fw 5'GCCGAGACTATCTGCACTAC

rev 5'ATGTCCAGCCTCAGAACTTC

p21 fw 5'GTGGCTCTGATTGGCTTTCTG

rev 5'CTGAAAACAGGCAGCCCAAG

Chk1 fw 5'AATGCTCGCTGGAGAATTGC

rev 5'TTGAGGGGTTTGTTGTACCATC

ATR fw 5'ACTCGCTGAACTGTACGTGG

Rev 5'TACCCTCAGGTGGGGTTTCA

Primers and probe for detection of intra- and extracellular HBV DNA:
5′-CCGTCTGTGCCTTCTCATCTG-3′ (sense),
5′-AGTCCAAGAGTCCTCTTATGTAAGACCTT-3′ (anti sense),
5-/56 FAM/CCGTGTGCA/ZEN/CTTCGCTTC ACCTCT GC/3IABkFQ/-3 (probe).

Antibodies

Goat anti-R2 (Santa Cruz Biotechnology (SCB) N18 SC-10844)

Mouse anti-Actin (Sigma A4700), Mouse anti-HA (Sigma)

Rabbit anti-PSMA4 was a kind gift of C. Kahana, Weizmann Institute of Science, Rehovot, Israel)

Rabbit anti-pChk1-ser345 (Cell Signaling Technology (CST) #2348), Rabbit anti-Chk1 (CST #2345)

Rabbit anti-E2F-1 (SCB C-20 SC-193;)

Rabbit anti-pATR-Thr1989 (Genetex GTX128145)

Mouse anti-ATR (SCB C-1 SC-515173).

Horseradish peroxidase–conjugated secondary antibody (Jackson)

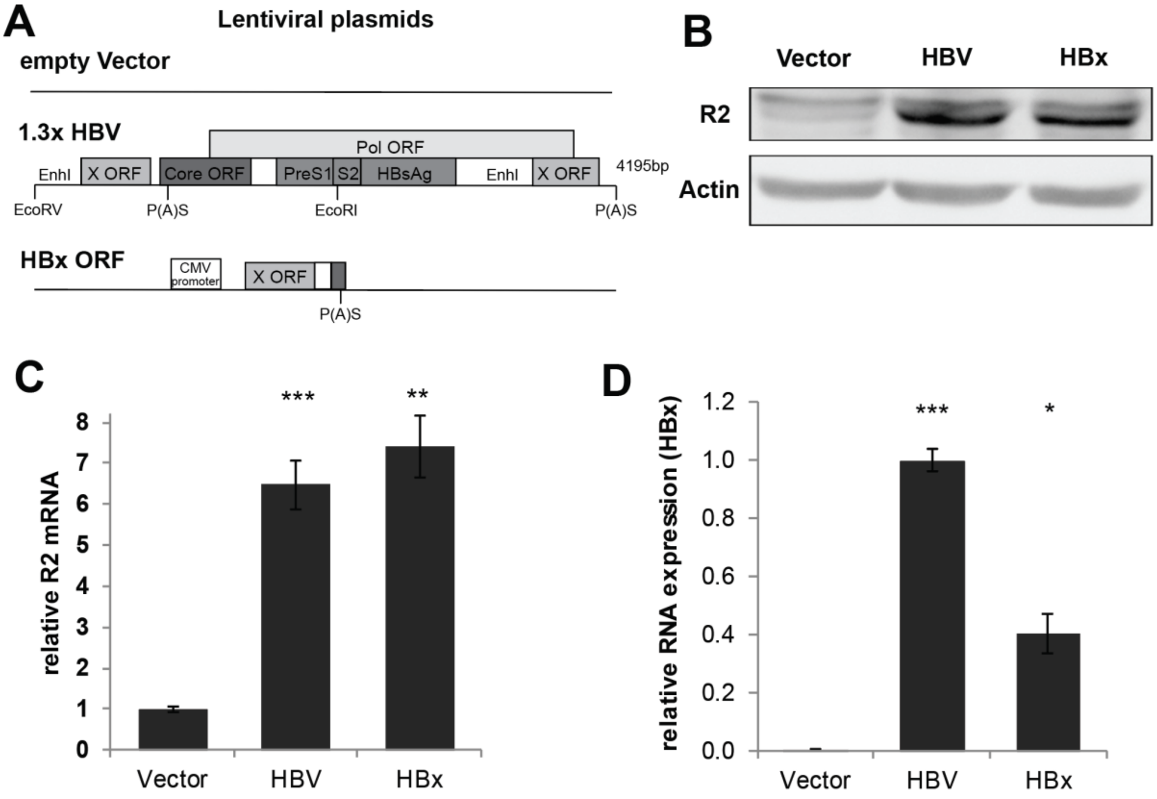

Figure S1. HBV and HBx upregulate RNR-R2. A) Schematic depiction of lenti-vectors (LV) used: empty vector without any HBV elements, the 1.3xHBV genome and HBx ORF under a CMV promoter. Quiescent HepG2 cells were transduced with the LV. B) RNR-R2 protein levels are shown in a representative Western blot. Actin was used as loading control. C) As in B, RNR-R2 mRNA levels were measured by qRT-PCR from three biological replicates. D) HBx RNA levels (HBx primer) were measured by qRT-PCR. Student's t-test was performed and samples were labeled * for $p < 0.05$, ** for $p < 0.01$ and *** for $p < 0.001$.

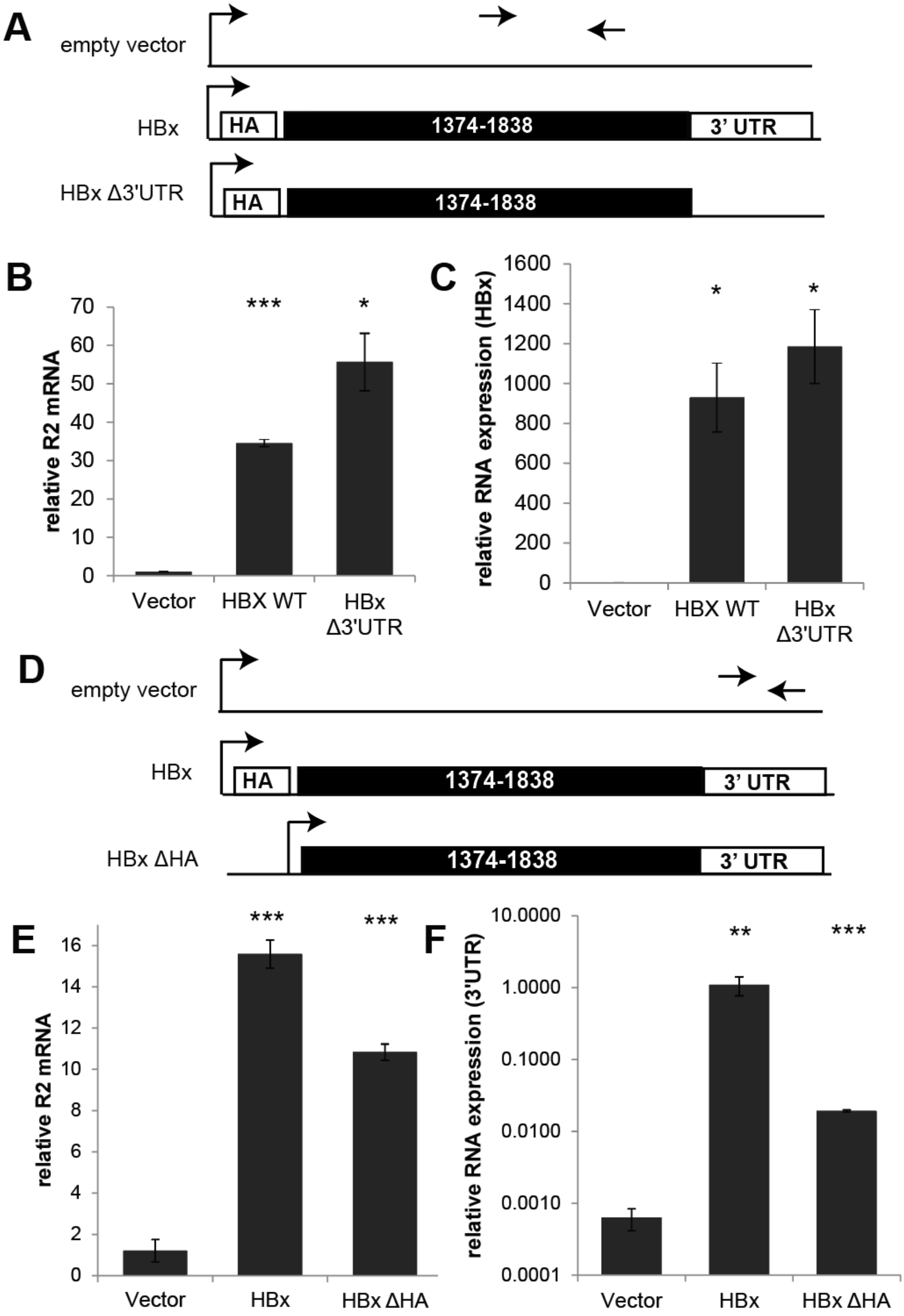

Figure S2. The endogenous 3′ UTR sequence is not required for RNR-R2 upregulation and neither is the 5′ flanking region. A) The endogenous HBV 3′ UTR was removed from the HA-X-3′UTR construct, as depicted in the illustration. B) The resulting construct was transduced into quiescent HepG2 cells, and fold RNR-R2 induction was measured by qRT-PCR. HA-HBx was used as positive control for RNR-R2 induction. C) Relative transduced RNA expression was measured

with a primer specific to the HBx sequence (primers indicated by forward and reverse arrow in A) by qRT-PCR. D) The HA sequence upstream to the HBx coding region was removed, as depicted in the illustration, and the resulting LV construct was transduced into quiescent HepG2 cells. E) Relative RNR-R2 mRNA levels were measured by qRT-PCR. HA-HBx was used as positive control and an empty lenti-vector as a negative control. F) Transduced RNA expression was measured by qRT-PCR, with primers from the shared HBX 3'UTR region, indicated as forward and reverse arrows in D. We depicted this graph with a logarithmic Y-axis, as the ΔHA-HBx contruct was expressed at lower levels than the HA-HBx but still exponentially and significantly higher than the empty vector. Student's t-test was performed and samples were labeled * for $p < 0.05$, ** for $p < 0.01$ and *** for $p < 0.001$.

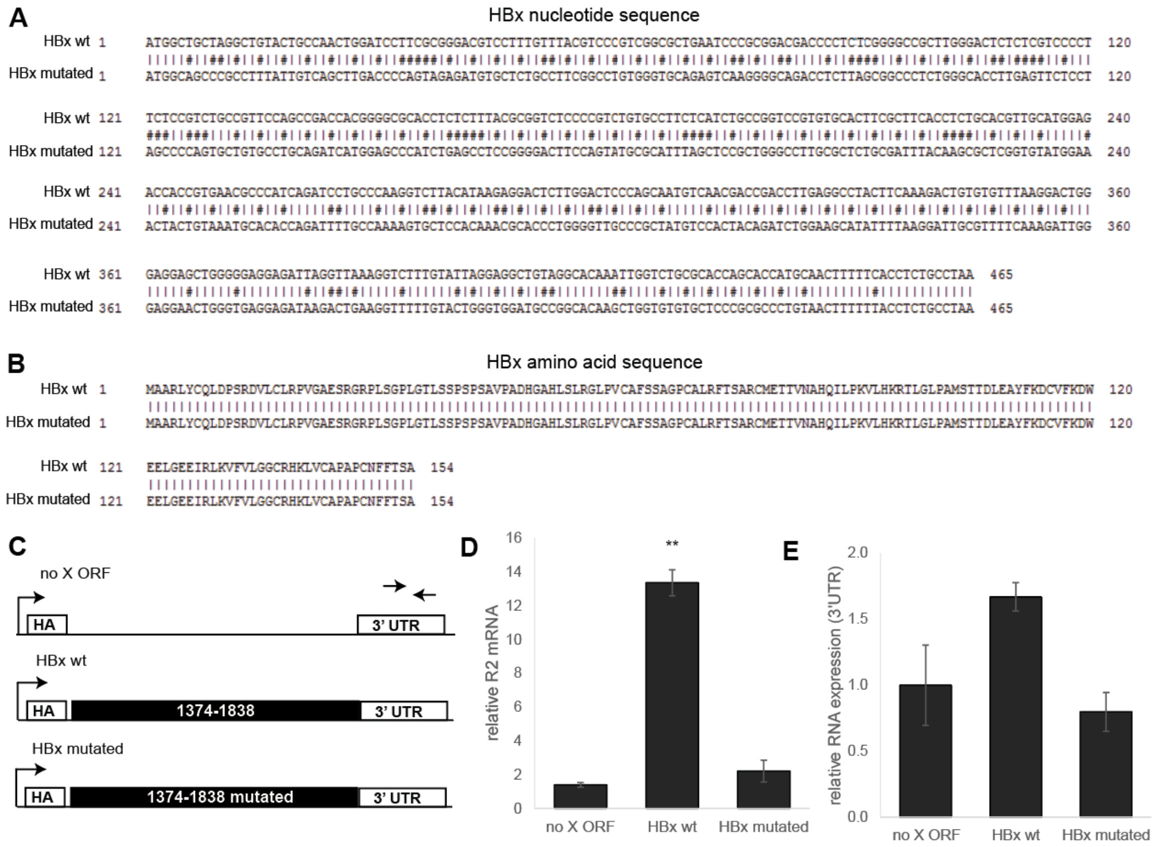

Figure S3. HBx nucleotide sequence mutation without changing HBx protein. A) Nucleic acid sequence alignment of HBx sequence (Genotype A, Adw2) and a sequence mutant. # represents mismatches. B) Amino acid sequence alignment of the sequences depicted in A. The amino acid sequence is the same. C) Schematic representation of lenti-plasmids used in this experiment: no-X contains HA and HBx 3'UTR sequence, but no HBx ORF, wt HBx and mutated HBx with the sequence in A. D and E) Quiescent HepG2 cells were transduced with constructs depicted in C. R2 mRNA (D) and HBx 3'UTR levels (E, primers indicated by forward and reverse arrows in C) were measured by qRT-PCR. Student's t-test was performed and samples were labeled ** for $p < 0.01$. Non-labeled samples are non-significant.

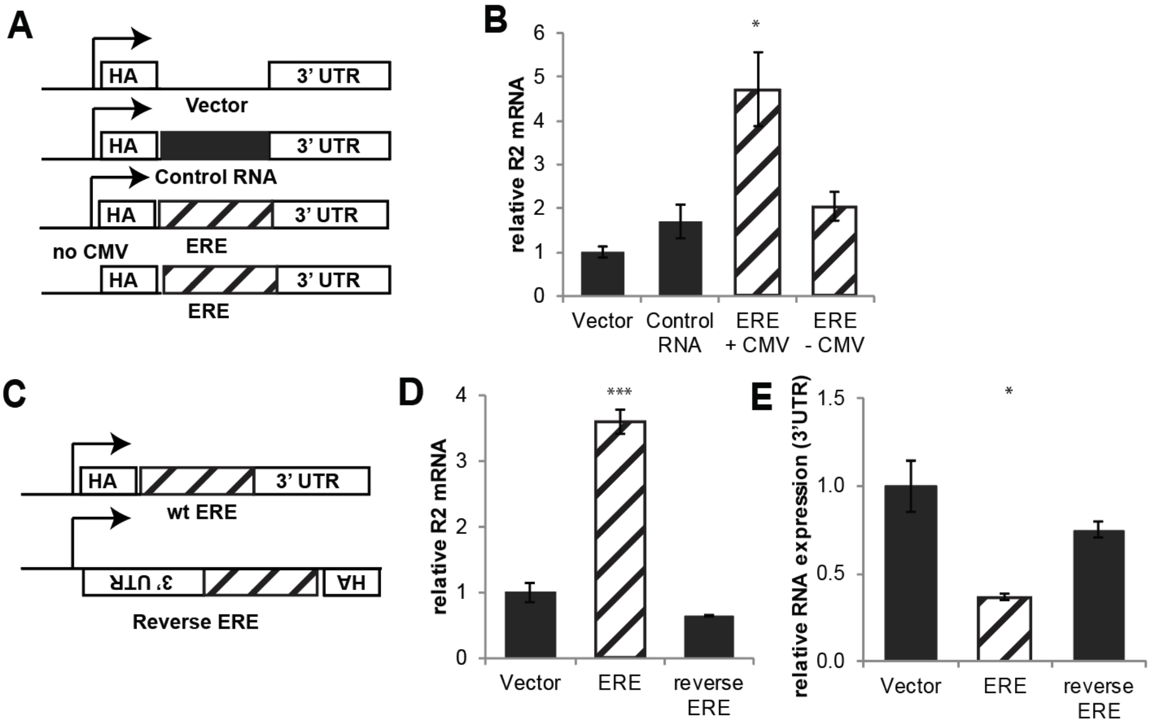

Figure S4. Functional ERE is a sense Pol-II transcript. A) The ERE fragment was cloned into a lentiviral construct with (+) or without (-) a CMV promoter. An empty LV and the control RNA fragment under CMV promoter were used as negative controls. B) Non-cycling HepG2 cells were transduced with the indicated constructs. Shown are relative RNR-R2 mRNA levels, measured by qRT-PCR from three biological replicates. Student's t-test was performed comparing all samples to the vector control, only ERE + CMV was significantly (*-$p<0.05$) changed. C) LV with ERE in sense and anti-sense (reverse-complement) orientation were constructed. D) Non-cycling HepG2 cells were transduced with the sense and reverse ERE. Relative RNR-R2 mRNA levels were measured by qRT-PCR from three biological replicates. T-test was performed comparing the samples with the vector control, only sense ERE changed significantly (***-$p<0.001$) E) Viral RNA (3'UTR) expression levels were measured by qRT-PCR. Student's t-test was performed and samples were labeled * for $p < 0.05$ and *** for $p < 0.001$. The non-labeled samples are non-significant.

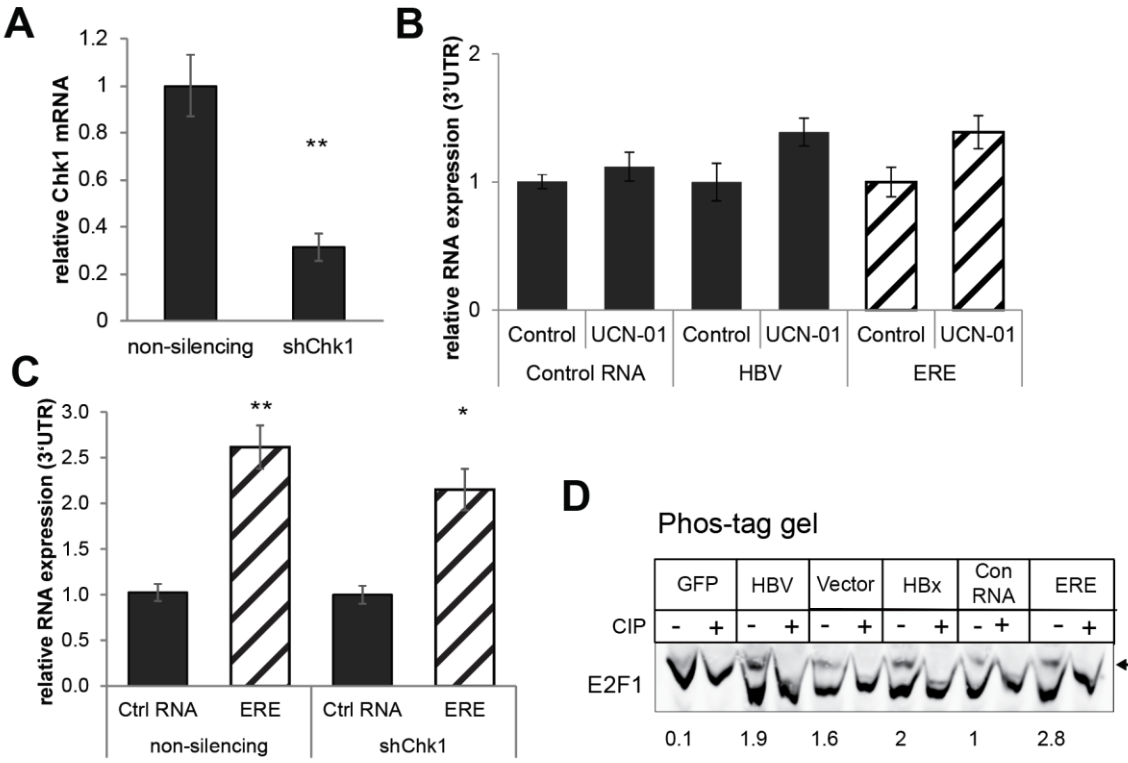

Figure S5. Chk1 is required for ERE activation or R2. A) Chk1 mRNA levels of HepG2 cells expressing shChk1 or non-silencing shRNA were measured by qPCR. B) Non-cycling HepG2 cells were transduced with the indicated constructs and treated with 1μM UCN-01, an inhibitor of Chk1 kinase activity, for 24h. Relative LV expression levels (3′UTR) were measured by qRT-PCR. C) Non-cycling HepG2 cells, expressing shChk1 or non-silencing shRNA were transduced with control RNA or ERE. Relative LV expression levels (3′UTR) were measured by qRT-PCR. D) Non-cycling HepG2 cells were transduced with the indicated LV constructs. The protein samples were treated with CIP or left untreated, and subjected to Phos-Tag SDS-PAGE gel, to separate the phosphorylated E2F1 forms. The arrow marks the phospho-E2F1 band. Student's t-test was performed and samples were labeled * for $p < 0.05$ and ** for $p < 0.01$. The non-labeled samples are non-significant.

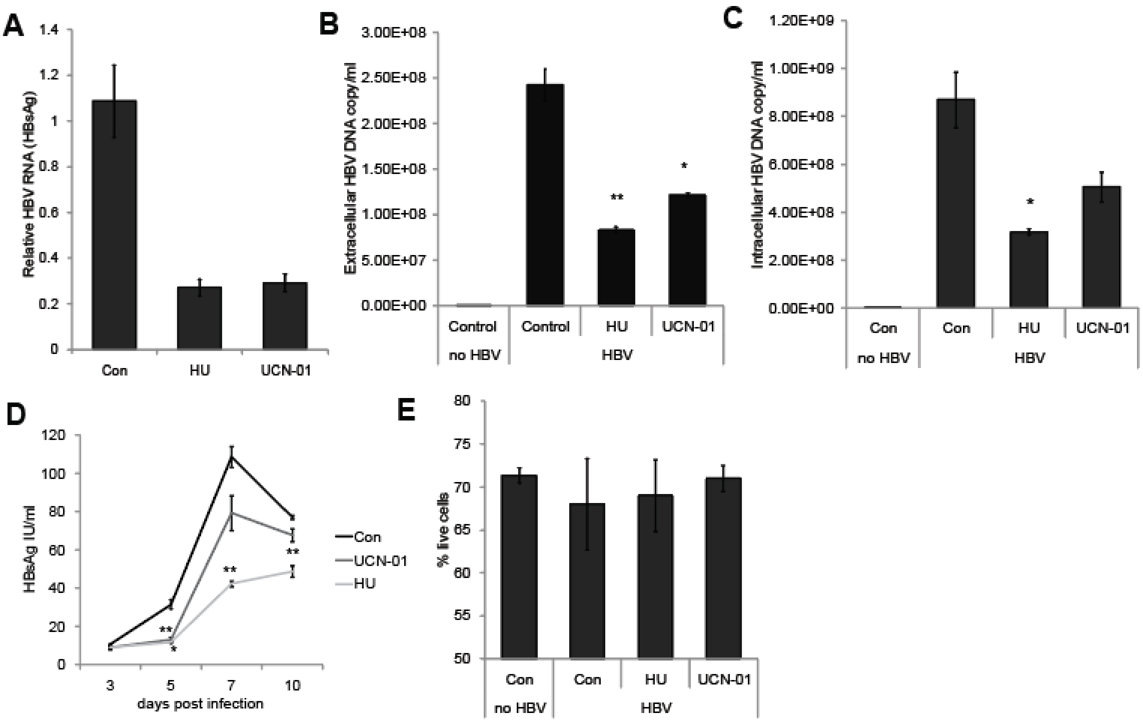

Figure S6. Inhibition of R2 and Chk1 by HU and UCN-01 affects viral replication but does not affect cell viability. PHH were infected with HBV or mock infected. After 24h, cells were treated with HU (15mM), UCN-01 (1µM) or vehicle (0.1%DMSO). HU was re-added every day, due to its very quick turnover, and UCN-01 was replaced every 2-3 days, with media change at days 3, 5, 7 and 10dpi. A) HBV RNA (HBsAg) 10 days post infection was measured by qRT-PCR. B) Extracellular HBV DNA 10 days post infection was measured by qPCR. C) Intracellular HBV DNA 10 days post infection was measured by qPCR D) HBsAg levels in the medium were measured by chemiluminescence immunoassay at the indicated time points. E) Live cells were measured 10 days post infection with HU and UCN-01 treatment. Student's t-tests were performed to compare the treated and untreated samples. * p-value<0.05, ** p-value<0.01.

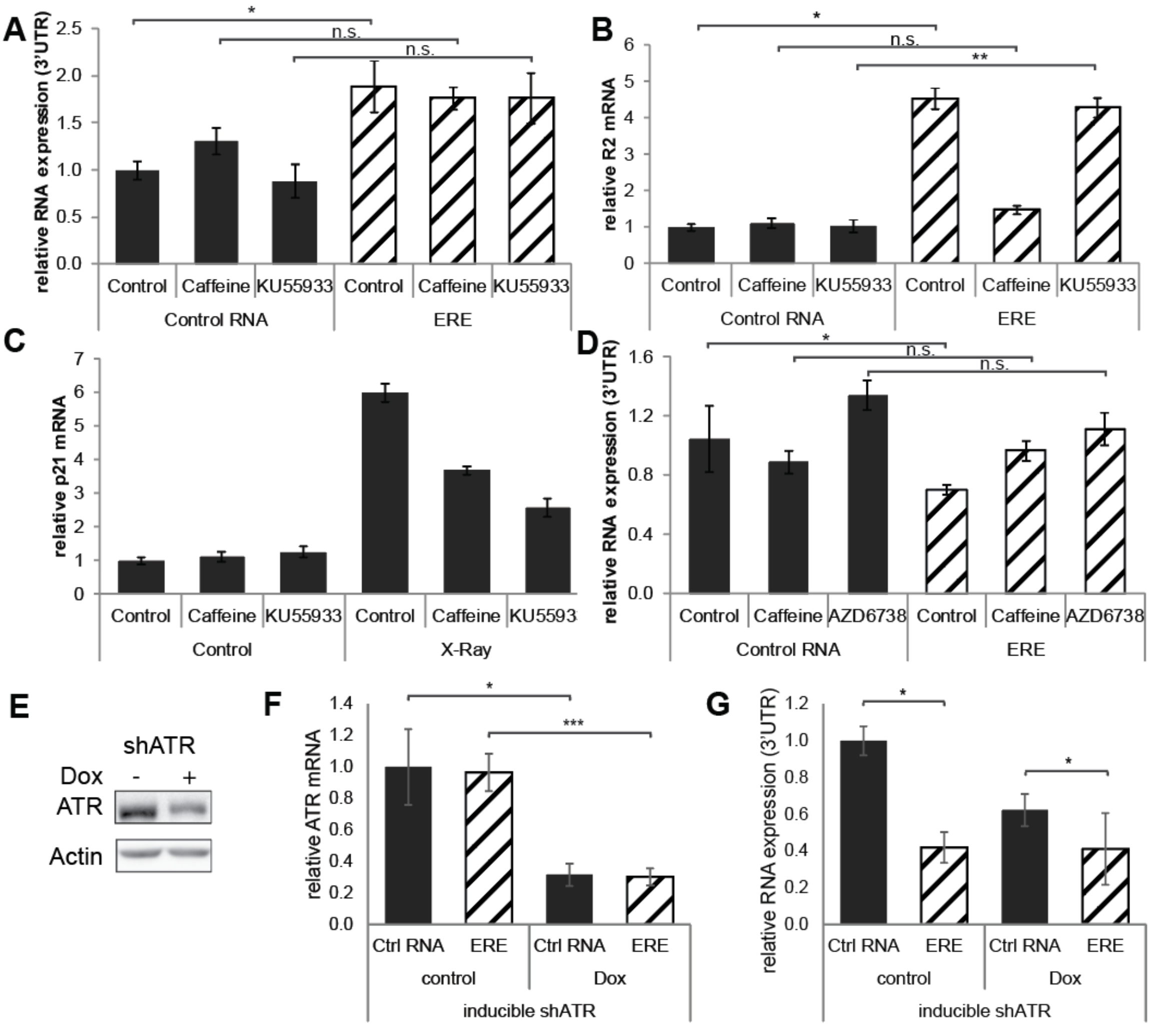

Figure S7. ATM inhibition does not affect ERE-mediated RNR-R2 induction. A) Non-cycling HepG2 cells expressing either ERE or the control RNA were treated with 2µM Caffeine (ATM/ATR inhibitor) or 1µM KU55933 (ATM inhibitor) for 24h. Relative RNR-R2 mRNA levels were measured by qRT-PCR. B) Transduced RNA (3'UTR) expression levels were measured by qRT-PCR to validate they remain unchanged following treatment with the indicated inhibitors. C) To validate the inhibitors, HepG2 cells were treated with the indicated inhibitors, irradiated with X-ray (5Gy), to induce DNA damage response, and harvested after 24h. Relative p21 mRNA levels were measured as an indication for DDR inhibition. D) Non-cycling HepG2 cells were transduced with LV-constructs containing ERE or control RNA. After 48h, cells were treated with 2µM Caffeine or 10µM AZD6738, a specific ATR inhibitor, or left untreated. Relative RNA (3'UTR) expression levels were measured by qRT-PCR. E) A HepG2-based cell line that expresses ATR shRNA under a Doxycycline (Dox)-inducible promoter was created. Cells were either treated with DMSO (control) or with Dox and harvested after 3d. WB analysis was performed with an ATR specific antibody and Actin was used for loading control. F and G) The cells were DMSO treated to be quiescent, transduced with ERE or control RNA and treated with 1ug/ml Dox for 3 days. ATR mRNA (F) and LV expression (3'UTR) (G) levels were measured by qPCR. Student's t-test was performed and samples were labeled * for $p < 0.05$, ** for $p < 0.01$ and *** for $p < 0.001$.

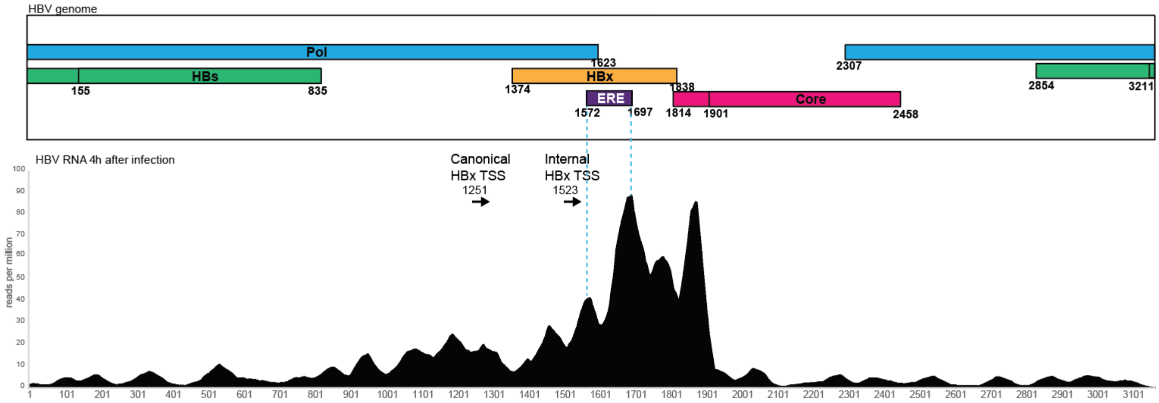

Figure S8. ERE sequence overlaps with RNA detected early in infection. Analysis of RNA-Seq data from GSE93153 uploaded by Niu et al. [21] of HBV infected PHH 4 hours post infection. The reads were aligned to the HBV genome and the canonical HBx TSS and reported new HBx internal TSS are shown [36,37].
